# Delivering high-quality multiple sclerosis care in Germany: challenges and regional models

**DOI:** 10.1186/s42466-026-00531-2

**Published:** 2026-09-07

**Authors:** Marc Pawlitzki, Niklas Huntemann, Lars Masanneck, Noah M. Werner, Julian Zimmermann, Thomas Postert, Christoph Lassek, Sven G Meuth, Klaus Gehring, Joachim Havla, Philipp Albrecht, Tjalf Ziemssen, Matthias Grothe

**Affiliations:** 1 Department of Neurology, University Hospital Münster, Albert-Schweizer Campus 1A, Münster, 48151 Germany; 2 Department of Neurology and Medical Faculty, University Hospital Düsseldorf, Düsseldorf, Germany; 3 Sauerlandklinik Hachen, Sundern, Germany; 4 Department of Neurology, Kamillus Klinik Asbach, Asbach, Germany; 5 Department of Neurology, St. Vincenz-Krankenhaus, Paderborn, Germany; 6 Neurological Group Practice, Kassel, Germany; 7 Neurozentrum am Klosterforst, Hanseaten-Platz 1, 25524 Itzehoe, Germany; 8 Institute of Clinical Neuroimmunology, LMU University Hospital, LMU Medizin, Ludwig- Maximilians-Universität München, Munich, Germany; 9 Department of Neurology, Kliniken Maria Hilf GmbH, Academic Teaching Hospital of the RWTH Aachen University Hospital, 41063 Mönchengladbach, Germany; 10 Center of Clinical Neuroscience, Department of Neurology, Faculty of Medicine, University Hospital Carl Gustav Carus, TUD Dresden University of Technology, Dresden, Germany; 11 Department of Neurology, University Hospital Greifswald, Greifswald, Germany

**Keywords:** multiple sclerosis, Germany, health services, neuroimmunology, quality indicators, telemedicine, shared care, digital health, de-escalation

## Abstract

Multiple sclerosis (MS) care in Germany has entered a phase in which therapeutic progress is increasingly constrained by delivery capacity. Earlier diagnosis, broader access to high-efficacy disease-modifying therapies (DMTs), and more refined monitoring strategies have improved the potential for favourable long-term outcomes. At the same time, these advances have increased the need for specialised expertise, infusion and monitoring infrastructure, rapid imaging access, structured safety procedures, and cross-sector coordination. Real-world evidence from Germany indicates a shift towards early high-efficacy treatment but also shows regional variation in treatment initiation, therapy intensity, and the proportion of people with MS who remain DMT-untreated. This expert-informed narrative review combines a non-systematic literature review with selected regional implementation examples from German MS care. This review synthesises key delivery challenges in German MS care and maps regional models whose underlying organisational principles may be transferable across healthcare settings, while their specific implementation depends on the local regulatory and reimbursement context. The central argument is that high-quality MS care should be understood not only as a therapeutic decision but as a system property: evidence-based treatment must be embedded in reliable pathways for diagnosis, monitoring, escalation, de-escalation, rehabilitation, and long-term support. Regional models such as ambulatory specialist care structures, hub-and-spoke networks, Germany´s inpatient MS complex treatment programme, delegated task models, telemedical consultation pathways, and digital monitoring infrastructures provide practical building blocks. Implementation at scale will require measurable quality indicators, financing of coordination work, formalised skill-mix models, digital interoperability, and explicit strategies for ageing and multimorbid MS populations. For neurological practice, the challenge is therefore not whether modern MS care is possible, but how to make it reproducible, safe, and equitable across regions.

## Introduction

Over the last decade, multiple sclerosis (MS) care has become simultaneously more effective and more operationally demanding. Earlier diagnosis and a broader spectrum of disease-modifying therapies (DMTs), including high-efficacy approaches, have improved the prospect of preventing inflammatory disease activity and disability accrual [1]. However, the same advances have increased the requirements for specialised expertise, infusion capacity, structured safety monitoring, access to magnetic resonance imaging (MRI), vaccination and infection-risk management, and coordination with radiology, rehabilitation, nursing, psychosocial services, and other specialties [2–4]. Thus, the impact of medical progress depends on whether the healthcare system keeps pace. Therapeutic innovation has limited value if the surrounding care system cannot provide timely access, processing, and exit routes. From a structural perspective, the key pillars are reimbursement and financing as well as regulation. From a care delivery perspective, the central elements are intersectoral collaboration and specialization within dedicated centres. The two levels are bridged by the training and specialization of all care-involved professionals, both clinical and non-clinical, as well as digitalization, which permeates all processes and is essential to both clinical practice and structural health system organization [5].

**Fig. 1:**
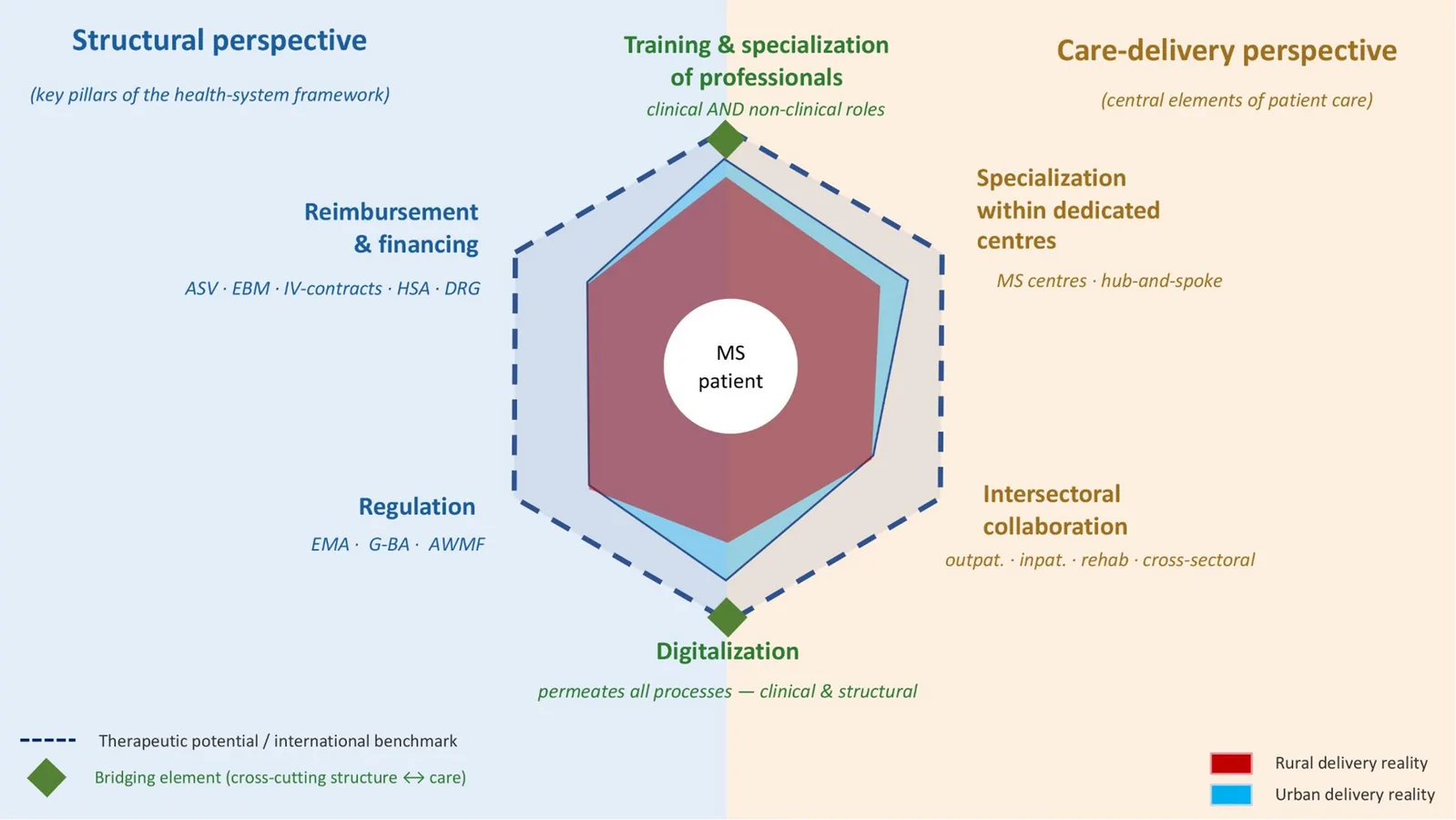
Schematic framework of determinants of MS care delivery across structural and care-delivery levels. The figure conceptualizes MS care delivery as the interaction between structural health-system determinants and care-delivery elements. Reimbursement and financing, together with regulatory frameworks, define the structural conditions under which MS care can be organized. Specialization within dedicated MS centres and intersectoral collaboration represent central elements of patient-facing care delivery. Training and specialization of clinical and non-clinical professionals, as well as digitalization, function as bridging elements that connect structural frameworks with practical care delivery. The concentric polygons illustrate the gap between therapeutic potential or international benchmarks, urban delivery reality, and rural delivery reality, emphasizing how regional differences in infrastructure, workforce, coordination, and digital maturity may influence whether available therapeutic options can be reliably implemented in routine care. Abbreviations: ASV, ambulante spezialfachärztliche Versorgung; AWMF, Arbeitsgemeinschaft der Wissenschaftlichen Medizinischen Fachgesellschaften; DRG, diagnosis-related group; EBM, Einheitlicher Bewertungsmaßstab; EMA, European Medicines Agency; G-BA, Gemeinsamer Bundesausschuss; HSA, Hochschulambulanz; IV, integrated care; MS, multiple sclerosis

This conceptual framework (Fig. 1) generates a testable expectation. If any of the four pillars (reimbursement and financing, regulation, intersectoral collaboration, or specialization within dedicated centres) is underdeveloped in a given region, or if either of the two bridging elements (training and specialization of all care-involved professionals, or digitalization) fails to span both levels, the distance between therapeutic potential and real-world delivery should widen there [6, 7]. German real-world evidence supports exactly this and shows that the gap is neither random nor solely clinical in origin. A survey among MS experts in Germany showed a gap between internationally proposed brain-health quality standards and routine feasibility, mainly due to insufficient resources, a deficit that maps onto the structural pillar of reimbursement and financing, as well as the bridging element of training and workforce specialization [8]. Pharmacy dispensing data indicate a shift towards early high-efficacy treatment since 2020, but with uneven regional implementation [9, 10]. Claims data further demonstrate regional differences in the proportion of DMT-untreated MS patients [11]. However, DMT non-treatment should not be equated with undertreatment, as treatment status may reflect disease course, age, comorbidity, contraindications, treatment preferences, or the absence of an indication for DMT, particularly in progressive disease. Complementing these outpatient and prescription-based data, a recent nationwide analysis of German inpatient and day-care cases from 2019 to 2024 showed that MS care is shifting away from inpatient settings in a regionally uneven manner, while the remaining inpatient population appears more complex and procedure-intensive [12].

These differences mirror variation in intersectoral collaboration and in the digital infrastructure required to organize complex care pathways across sectors. Taken together, these findings suggest that variation in treatment is not explained by clinical preference alone. It reflects differences in the structural pillars, in the care-delivery elements, and in how well training and digitalization actually bridge the two.

In this context, high-quality MS care refers to care that is clinically appropriate, safe, equitable, coordinated, and responsive to the individual needs and disease course of people with MS. It requires timely access to appropriate expertise and treatment, structured monitoring, effective multidisciplinary and cross-sectoral coordination, and the infrastructure and workforce needed to implement evidence-based care consistently across settings. Quality is therefore understood not solely as the appropriateness of individual therapeutic decisions, but as a property of the care system as a whole.

The aim of this narrative review is therefore to synthesise delivery challenges in MS care in Germany and to map regional models that address them. The focus is on transferable building blocks and policy levers that could improve quality, safety, and equity at scale.

## Methods

This narrative, expert-informed implementation review was initiated by a structured expert discussion held in February 2026, during which experts in MS care discussed documented regional gaps in Germany and shared regional solutions from routine practice. Experts were selected based on their established clinical, academic, and/or health-services expertise in MS care and related areas, including regional care coordination, multidisciplinary care, digital health, and health-services organisation. The meeting results were recorded and used to define the main thematic domains of this paper.

In addition, a non-systematic review of the relevant scientific and health-policy literature was performed, with searches conducted in PubMed and covering the period up to June 2026. The search focused on German real-world evidence, quality indicators, digital care pathways, regional access analyses, and workforce models. Targeted searches of health-policy, professional-society, and institutional sources were undertaken where required to identify information on specific regional implementation models and regulatory frameworks. Where published evidence was unavailable, information on the organisation and volume of care at individual institutions was based on data provided by the respective institutions through participating experts.

Regional models were selected purposively to illustrate different approaches to the care gaps identified during the expert discussion. Selection was guided by the relevance of each model to a specific delivery challenge, its implementation in routine care, and the availability of sufficiently detailed information from published literature, institutional or health-policy sources, or expert knowledge. Given the authors’ involvement in several regional and digital care initiatives, some examples were drawn from the authors’ own centres and prior work and, where available, complemented by models reported or evaluated independently. The selected examples are intended to illustrate a range of organisational and structural approaches and should not be interpreted as a systematic, representative, or comparative assessment of MS care models or provider quality in Germany.

The assessment of evidence, maturity, and transferability was descriptive and did not follow formal rating criteria. Evidence reflects the strength and availability of published or otherwise documented support for a given care approach; maturity describes the extent to which an approach has progressed from pilot or development to routine implementation; and transferability reflects the authors’ expert assessment of its applicability to other settings, considering organisational requirements, infrastructure, workforce, regulatory conditions, and reimbursement. The evidentiary basis of individual examples varied and included peer-reviewed publications, formal or ongoing evaluations, routine-care or institutional data, and expert-informed descriptions where published evidence was unavailable. These sources were not considered equivalent, and the resulting assessments should therefore be regarded as preliminary rather than as formal evidence grading.

### Core challenges in German MS care

#### Regional access gaps and capacity constraints

Regional differences in MS treatment and access suggest that the availability of specialised services and workflow capacity shapes care in Germany. In a claims analysis covering 2017 to 2022, nearly half of the patients were not treated with DMTs during the observation period, with rates between 37.5 and 54% of untreated patients summarized by the first postal region of residence [11]. Access to specialized health care is also regionally different. A German travel-time analysis showed that 64.2% of the population can reach a certified MS centre within 30 min by car and 96.5% within 60 min [13]. Compared with rare neuroinflammatory diseases, this coverage is relatively favourable. However, for patients with impaired mobility, long travel distances remain clinically meaningful. Travel burden increases caregiver strain, may reduce adherence to monitoring, and can delay decisions in patients requiring complex therapy changes or relapse management [14].

Interestingly, international quality standards for brain-health focused MS care have proposed timeframes for reporting new symptoms, diagnostic work-up, treatment decisions, and monitoring [15]. However, a German expert survey found that several of these timeframes were not feasible in routine practice even in urban regions because of limited resources [8]. Referral to other services and cognitive screening were also perceived as difficult due to insufficient local infrastructure [8]. These data suggest that access should not only be evaluated by distance to specialist centres but also by operational capacity once patients enter the system.

#### Early high-efficacy treatment versus real-world feasibility

Real-world data indicate that German MS care is moving towards earlier high-efficacy strategies [9, 10]. This is consistent with evidence-based treatment concepts in relapsing MS [16], but its success depends on local deliverability. Early high-efficacy treatment requires structured risk stratification, safety laboratory monitoring, vaccination review, infection-risk management, reliable MRI capacity, and rapid escalation pathways for adverse events or breakthrough activity [4, 17]. The burden of monitoring should already be considered when new therapies and new care pathways are developed, because therapies that are mechanistically compelling may still fail in routine care if monitoring pathways are unrealistic [18, 19]. Consequently, evidence-based intent and practical deliverability may diverge in understaffed settings, limited infusion capacity, or weak interfaces between outpatient neurology, hospital-based centres, and radiology.

#### Ageing, comorbidity, and treatment exit strategies

With increasing age, multimorbidity, polypharmacy, frailty, disability, and the need for community-based or nursing-home support is increasingly central to therapeutic decision making [20–22]. Each of these factors can alter the benefit-risk balance of long-term immunotherapy for some patients, leading to a higher clinical and structural relevance of structured frameworks for de-escalation or discontinuation [23–28]. This also reflects a conceptual shift in MS care: treatment outcomes are increasingly shaped not only by the choice and timing of DMTs, but also by structured symptomatic management, multidisciplinary neurological complex treatment, rehabilitation, and, where appropriate, geriatric, and palliative care [26, 29]. For patients with progressive disability, multimorbidity, or complex functional impairment, such multidisciplinary care enables comprehensive reassessment of neurological status, optimisation of disease-modifying and symptomatic therapies, coordinated rehabilitation planning, and structured transition back into long-term outpatient care.

#### Fragmentation, workforce and the limits of coordination

MS care is inherently multi-sectoral, spanning outpatient neurology, hospital-based neuroimmunology, MS care units, radiology, rehabilitation, nursing services, social work, psychological support, general practice, and patient organisations [30–32]. In many regions, however, the structures needed to navigate and streamline this complexity - such as virtual case discussions, shared documentation, rapid specialist advice, and clearly defined multiprofessional workflows - remain difficult to implement in routine care. As a result, coordination work becomes clinically essential but often remains poorly visible in staffing models and reimbursement. Multidisciplinary inpatient neurological complex treatment programmes exemplify this challenge. Their effectiveness depends not only on specialised neurological expertise but also on structured coordination between physicians, specialised nurses, therapists, neuropsychologists, social workers, and rehabilitation services. Much of this interdisciplinary work consists of communication, treatment planning, goal setting, and discharge coordination rather than discrete billable procedures, making its resource requirements insufficiently visible in conventional reimbursement models [33, 34].

At the same time, workforce shortages are not confined to neurologists. Complex MS pathways also depend on MS nurses, advanced medical assistants, physician assistants, radiology staff, rehabilitation professionals, and administrative coordinators. Limited availability of these trained non-physician roles restricts the capacity to scale monitoring, education, triage, and documentation. This is well illustrated by relapse care: a substantial proportion of steroid treatment for acute relapse could be delivered in an outpatient setting, but if the required outpatient structures, delegated roles, and reimbursement pathways are lacking, patients may still be treated as inpatients even when outpatient care would be clinically adequate and less costly. Structured neuroimmunology training pathways, clear delegation rules, and competency tiers should therefore be understood not as peripheral organisational details, but as core requirements for maintaining care quality under capacity constraints [30].

Table 1 translates these interdependent challenges into measurable indicators and scalable implementation pathways, providing a practical bridge from structural diagnosis to implementation and highlighting where MS care can be measured, responsibilities assigned, and equity improved across regional settings.

**Table 1:** Delivery challenges, indicators, evidence and scalable solution pathways

Challenge	Measurable indicator	Solution pathway	Evidence	Main barrier / stakeholder	Time-to-implement
Capacity constraints	Median time-to-DMT initiation; % MRI regular follow-ups	Remote monitoring; digital coordination layer with PROs, reminders, escalation triggers	Medium	Reimbursement; statutory health insurance	Medium (1–3 yrs)
Access gaps (rural vs. urban)	Mean travel time to certified MS centre	Telemedicine; local shared-care partners; planned escalation slots at hub	Medium	Broadband; reimbursement for video consult	Short (< 1 year)
Quality inequity across regions	DMT rate per region; untreated proportion; rehabilitation access	Consensus quality indicators; equity dashboards by federal state	Low–Medium	Data interoperability; G-BA, KKNMS, MS-Registry	Medium (1–3 yrs)
Early high-efficacy treatment	% MS patients on high-efficacy DMT within 6 months of diagnosis	Hub-and-spoke shared-care agreements with defined roles, monitoring, escalation rules	High	Workforce in spokes; MS-centres + office-based neurology	Medium (1–3 yrs)
Monitoring burden	Adherence to safety-lab schedule; missed visit rate	Task delegation to trained non-physician staff; competency tiers	Medium	Curriculum, supervision rules; BÄK, BVDN, KBV	Medium (1–3 yrs)
Ageing MS population	% MS patients ≥ 55 yrs; multimorbidity index; polypharmacy prevalence	Structured de-escalation pathways; symptom-oriented care; palliative neurology	Low–Medium	Evidence on discontinuation; specialist centres + geriatrics	Long (> 3 yrs)
Comorbidity & polypharmacy	Drug-drug interaction screens; medication review rate	Structured medication review by MS nurse / clinical pharmacist	Medium	Time and reimbursement; office-based neurology, pharmacy	Short (< 1 year)
Fragmentation	Number of virtual MS boards; cross-sector documents shared	Virtual case discussions; shared documentation; reimbursed coordination	Low	Reimbursement of coordination work; G-BA, GKV-SV	Long (> 3 yrs)
Workforce beyond physicians	MS-nurses per 100 MS patients; PA/AMA per practice	Specialised nurse education; AMA / PA team-based workflows; Papenburg-style delegation	Medium	Training pathways; chambers, universities, MS centres	Medium (1–3 yrs)

Table 1 Delivery challenges, measurable indicators and scalable solution pathways for multiple sclerosis care. The table outlines key structural and clinical challenges affecting the delivery of MS care and links them to potential metrics for monitoring system performance, proposed solution pathways, current level of supporting evidence, main implementation barriers, relevant stakeholders and estimated time-to-implementation. It is intended to provide a pragmatic framework for identifying actionable gaps in MS care and prioritising scalable interventions across outpatient, specialist-centre and cross-sector care settings. Abbreviations: AMA, ambulatory medical assistant; BÄK, German Medical Association (Bundesärztekammer); BVDN, Professional Association of German Neurologists (Berufsverband Deutscher Nervenärzte); DMT, disease-modifying therapy; G-BA, Federal Joint Committee (Gemeinsamer Bundesausschuss); GKV-SV, National Association of Statutory Health Insurance Funds (GKV-Spitzenverband); KBV, National Association of Statutory Health Insurance Physicians (Kassenärztliche Bundesvereinigung); KKNMS, Disease-Related Competence Network Multiple Sclerosis (Krankheitsbezogenes Kompetenznetz Multiple Sklerose); MRI, magnetic resonance imaging; MS, multiple sclerosis; PA, physician assistant; PRO, patient-reported outcome; yrs, years.

### Scalable solution pathways and regional implementation models

Against this background, the regional care models discussed in this manuscript are unified by a common design principle: they aim not to replace specialist neurological expertise, but to redistribute it more effectively across the care pathway, ensuring that limited specialist resources are allocated to the clinical constellations where they add the greatest value. Importantly, transferability should be understood at the level of organisational principles rather than as direct replication of the German implementation models. While approaches such as hub-and-spoke networks, skill-mix, delegated care, structured coordination, and digital pathways may be applicable across healthcare systems, their concrete implementation depends on local regulation, reimbursement structures, workforce models, and sectoral organisation.

In these models, complex diagnostic and therapeutic decisions remain anchored in specialised centres, while structured monitoring, longitudinal follow-up, and supportive care are increasingly delivered closer to patients. Key components include cross-sectoral care frameworks, regional integration of specialist expertise, skill-mix approaches and task delegation, digital care pathways, and clearly defined interfaces with rehabilitation, palliative care, and patient organisations [35]. In this way, network-based care may reduce direct, indirect, and intangible costs by lowering disease burden, avoiding duplicate examinations, accelerating diagnosis, and shifting avoidable inpatient activity into more appropriate outpatient pathways [36, 37]. Table 2 summarises the regional implementation models discussed in this review, grouping them according to the principal care-delivery challenge they address and highlighting their implementation maturity and potential transferability.

**Table 2:** Regional MS care models grouped by delivery category

Category	Delivery problem addressed	Concrete regional implementations	Maturity & transferability
Digital: first-contact triage	Shorten time from suspected MS to specialist evaluation in low-density regions, where waiting times for neurology appointments delay diagnosis.	Innovation Fund TENEAM project (funding ID 01NVF24001). Lead: Charité University Medicine Berlin, Carl Thiem University Medicine Cottbus, and University Medicine Greifswald. A structured, telemedicine-supported initial care pathway for neurological patients, such as those with suspected MS, in Brandenburg and Mecklenburg-Western Pomerania.	Pilot under controlled evaluation. Transferability high to other rural areas once a reimbursement pathway exists.
Digital: rural follow-up and consultation	Bring MS expertise to clinics that cannot recruit MS-experienced neurology locally.	LMU Munich telemedicine network: urban hub providing structured telemedical consultation to surrounding rural clinics where MS expertise was lacking.	Routine. Transferability high; depends on stable interfaces between hub and partner clinics.
Day clinic for MS diagnostics and complex therapy	Bridge the gap between outpatient and inpatient care through structured short-stay programmes, without the resource burden of a full inpatient stay (§ 115e SGB V).	University Medicine Greifswald: institution-reported approximately 200 complex diagnostic or therapeutic procedures per year.	Routine at many MS centres but heterogeneously implemented. Transferability high; depends on hospital infrastructure and reimbursement clarification.
Multidisciplinary neurological complex treatment	Provide structured inpatient multidisciplinary care for patients with complex diagnostic, therapeutic, or functional needs requiring coordinated neurological reassessment, optimisation of disease-modifying and symptomatic therapy, interdisciplinary treatment, and discharge planning.	Dedicated MS hospitals such as Kamillus Klinik Asbach and Sauerlandklinik Hachen provide structured multidisciplinary neurological complex treatment with integrated medical reassessment, treatment optimisation, and interdisciplinary care pathways for patients with complex MS.	Routine in specialised neurological hospitals. Transferability medium; requires multidisciplinary neurological infrastructure and appropriate reimbursement.
Inpatient complex and palliative care	Reduce symptom burden, caregiver strain, and psychosocial distress for patients at advanced stages of the disease, including severe impairment, complex symptom burden, and end-of-life situations, through early, integrated outpatient or home-based interventions.	University Medicine Greifswald operates a dedicated neurological palliative care unit serving patients with advanced neurological diseases including MS. Care prioritises realistic goal-setting, advance care planning, and compassionate communication, supported by symptom optimisation, psychosocial support, and social care embedded for both patients and caregivers.	Routine. Transferability medium; requires tertiary infrastructure including an integrated palliative care network and stable therapeutic capacity.
MZEB: comprehensive care for adults with severe disability	Provide an integrated medical, therapeutic, and psychosocial service for adults with severe MS-related disability, for example > 50% GdB, who are underserved by standard outpatient pathways because of mobility, communication, cognitive, or somatic comorbidity. Medizinische Zentren für Erwachsene mit Behinderung (MZEB; § 119c SGB V) are explicitly designed for this group.	MZEB at University Medicine Greifswald. Team led by neurologists, with multidisciplinary collaboration across neurology, palliative care, and allied health services within the university medical centre.	Routine in a growing number of regions. Transferability medium; requires KV-level § 119c SGB V authorisation and a hospital willing to host the structure.
ASV and hospital-based outpatient structures	Operationalise cross-sector specialist outpatient care at scale, with division of labour between office-based neurology and hospital-based specialist structures.	St. Vincenz Hospital Paderborn: institution-reported approximately 2,000 outpatient contacts per year, 8 infusion stations serving approximately 350 MS patients receiving DMT, and an inpatient unit under development with a projected capacity of approximately 500 patients per year. The model also includes consultations for office-based neurologists and GPs. MS specialist practice Kassel: institution-reported approximately 6,000 MS patients in standard outpatient care plus approximately 1,100 within ASV-MS. Maria Hilf Clinic Mönchengladbach: institution-reported approximately 1,000 MS patients, approximately 50 incident diagnoses per year, and ASV authorisation in a mid-sized town of approximately 300,000 inhabitants.	Routine, currently scaling. Transferability high with increasing numbers of ASV structures.
Continuing education, networking, and patient engagement	Operationalise the bridging element of training and specialisation of all care-involved professionals identified in Fig. 1 and sustain regional knowledge transfer.	Paderborn: regular workshops and seminars for neurologists, GPs, and MS nurses. MS-Net Mönchengladbach: monthly immunoboards and roundtables with regional speakers, formal collaboration with patient organisations, and investigator-initiated clinical research, including a study on sleep disorders, fatigue, and cognitive impairment in MS. Kamillus Klinik Asbach: neurological hospital operated by the North Rhine-Westphalia chapter of the German Multiple Sclerosis Society (DMSG), facilitating close collaboration between specialised neurological care, patient education, counselling, and local self-help networks.	Routine. Transferability high; low capital requirement but depends on individual engagement and sustained regional coordination.

Table 2 summarises selected regional implementation models for MS care in Germany and groups them according to the main delivery problem they address, including digital first-contact triage, rural telemedical consultation, day-clinic pathways, multidisciplinary neurological complex treatment, inpatient complex and palliative care, MZEB-based care for adults with severe disability, ASV and hospital-based outpatient structures, and continuing education, networking, or patient-engagement formats. For each category, the table links the underlying care gap to concrete regional examples, their current maturity, and their estimated transferability to other settings. Maturity refers to the extent to which an approach has progressed from pilot or development to established routine implementation; transferability reflects an expert-informed assessment of the extent to which its underlying organisational principles could be applied in other settings. These classifications are descriptive and should not be interpreted as formal or validated ratings. Institution-level care activity data without a corresponding literature citation are based on information reported by the respective institutions through the participating experts. Abbreviations: ASV, ambulatory specialist care (Ambulante spezialfachärztliche Versorgung); DMSG, German Multiple Sclerosis Society (Deutsche Multiple Sklerose Gesellschaft); DMT, disease-modifying therapy; GdB, degree of disability (Grad der Behinderung); GP, general practitioner; KV, Association of Statutory Health Insurance Physicians (Kassenärztliche Vereinigung); LMU, Ludwig Maximilian University of Munich; MS, multiple sclerosis; MZEB, medical centre for adults with disabilities (Medizinisches Zentrum für Erwachsene mit Behinderung); SGB V, German Social Code Book V (Sozialgesetzbuch V).

#### Structural entry points: ASV-MS and complex care pathways

In Germany, the German Federal Joint Committee (Gemeinsamer Bundesausschuss, G-BA) introduced ambulatory specialist care for MS within the framework of Ambulante Spezialfachärztliche Versorgung (ASV). ASV aims to provide specialised, interdisciplinary outpatient care across medical specialties, allowing hospital physicians and office-based physicians to collaborate under a common framework. Physicians must meet defined qualification requirements and apply for ASV accreditation; patients and referring physicians can use ASV directories to identify authorised providers [38].

Such a model strengthens continuity within smaller MS teams while embedding them in a broader interdisciplinary network that operationalises intersectoral collaboration: MS-experienced radiology with rapid access to therapy-specific MRI sequences, ophthalmology for visual-pathway monitoring, neuropsychology for cognitive assessment, urology and pain medicine for symptom management, rehabilitation, and palliative neurology for advanced disability. The practical value of such a network depends less on the number of disciplines involved than on the quality of the interfaces between them: shared documentation, standardised referral pathways, and explicit response-time commitments are what turn a list of specialties into a functioning care pathway.

Current implementation barriers include complex admission criteria, unclear national regulations, and outdated professional requirements. For example, the absence of key diagnostic procedures prevents the reimbursement of important quality instruments and their integration into healthcare provision. These include standardised cognitive tests, fatigue questionnaires, optical coherence tomography, and modern biomarkers such as serum neurofilament light chain. To address these challenges, the Disease-related Competence Network Multiple Sclerosis (KKNMS) and the German MS Society (DMSG) call for reform in a position statement, including standardising state committee procedures, removing irrelevant requirements from the service catalogue, and incorporating modern diagnostic procedures. Furthermore, systematic quality assurance is needed, ideally in cooperation with registries such as the German MS Registry of the DMSG [39].

Complex care pathways represent a complementary structural entry point. For patients with higher disability, complex symptom burden, or diagnostic and therapeutic needs that exceed standard outpatient capacity, specialised short-stay and day-clinic structures can help bridge the gap between office-based neurology and full inpatient admission. Such programs may combine diagnostic work-up, including lumbar puncture, MRI and evoked potentials, supervised initiation or optimization of DMTs, complex infusion treatments, multidisciplinary review, structured therapy counselling, and coordination with rehabilitation or community-based support. In Germany, one established implementation of such pathways is multidisciplinary neurological complex treatment. These structured inpatient programmes are embedded within specialised neurological departments and complement ambulatory specialist care by combining comprehensive neurological reassessment with complex therapeutic decision-making. In addition to targeted multidisciplinary therapies, they provide a dedicated framework for resolving complex diagnostic questions, optimising disease-modifying and symptomatic treatment, coordinating interdisciplinary management, and planning transition back into long-term outpatient care. Accordingly, the primary objective is not long-term functional restoration, as in neurological rehabilitation, but comprehensive medical reassessment and optimisation of specialised neurological care in parallel with targeted interdisciplinary therapy. This model has been implemented in several dedicated neurological hospitals in Germany, including the Kamillus Klinik Asbach and Sauerlandklinik Hachen, where multidisciplinary neurological complex treatment programmes complement ambulatory specialist care and rehabilitation within integrated regional care pathways [40–42].

For MS patients with severe disability, additional structures are needed that explicitly address mobility limitations, communication barriers, cognitive impairment, somatic comorbidity, and psychosocial needs [43–45]. Medizinische Zentren für Erwachsene mit Behinderung (MZEB; § 119c SGB V) provide one such framework by integrating medical, therapeutic, nursing, and social-care expertise for adults with complex disability who are often underserved by conventional outpatient pathways [46]. Where available, MS-specific pathways within MZEB structures could complement specialised neurological centres and provide a formal interface with rehabilitation, community services, and palliative care. At the University Hospital Greifswald, a dedicated neurological palliative care unit further complements this approach for MS patients with severe disability, a service currently provided by only a few centres and directed at an underserved part of the MS-care pathway.

#### Regional integration of specialised MS expertise

Specialised MS expertise can be integrated regionally through different models, depending on local geography, workforce availability, and existing outpatient or hospital-based structures. The common aim is to make specialist input accessible beyond the walls of large university centres while maintaining clear pathways for escalation, monitoring, and complex decision-making [47].

Low-density regions require models that extend specialist expertise without requiring every site to build full specialist capacity. German health care data analysis demonstrated that, despite unrestricted access to DMTs, patients with lower socioeconomic status were significantly less likely to receive high-efficacy treatment and experienced less favorable treatment patterns, indicating persistent socioeconomic disparities in MS care. These findings suggested that factors beyond healthcare coverage contribute to inequities in treatment decisions and access to optimal care [48]. Based on these results, at LMU Hospital Munich, a regional Neuroimmunology Network was founded (NeNET). This is a Hospital-based Network of neurological departments established through formal cooperation agreements. It provides structured neuroimmunological consultations for complex cases, facilitating access to specialist expertise, supporting diagnosis and treatment decisions, and promoting standardized, high-quality care for patients with neuroimmunological disorders across south Bavaria. At St. Vincenz Hospital Paderborn, an authorised outpatient MS-care structure handles approximately 2,000 outpatient contacts per year, supported by eight infusion stations for around 350 MS patients on DMTs; an inpatient unit projected at about 500 patients per year is currently under development. The Paderborn model is explicitly outward-facing: consultations for office-based neurologists and general practitioners are complemented by sustained public-relations work and active engagement with regional politics, which proved decisive for translating a local solution into a regional reference.

Other regional models illustrate how high-volume outpatient practice and specialist-level structures can be combined. The MS specialist practice in Kassel treats approximately 1,100 MS patients within ASV-MS, demonstrating that office-based care and specialist-level structures can coexist at scale. In Mönchengladbach, a mid-size town of about 300,000 inhabitants with approximately 1,000 MS patients and around 50 incident diagnoses per year, the Maria Hilf clinic combines ASV with the MS-Net care and networking project, creating a recurring forum for regional collaboration and shared care.

#### Teaching, skill-mix and delegated care models

Capacity can be expanded by shifting clearly defined tasks to trained non-physicians, including MS nurses, advanced medical assistants, and physician assistants [49]. Suitable tasks include safety laboratory checks, vaccination and infection-risk checklists, patient education, adherence support, structured triage, patient-reported outcome capture, and quality-indicator driven documentation [50].

Whether such redistribution succeeds or merely shifts workload depends on the training and specialisation of all care-involved professionals as bridging elements identified in Fig. 1. In practice, this requires formal curricula, defined supervision rules, competency tiers tied to specific responsibilities, and measurable quality indicators that make the effect of delegation visible [47, 51].

The Papenburg model illustrates how regional care for people with chronic neurological conditions such as MS can be strengthened through structured qualification, delegation, and network support. Papenburg is located in a comparatively rural region with quantitatively limited access to both general practitioners and medical specialists, a challenge that is expected to intensify in the coming years due to demographic change. Against this background, a model was established that combines structured qualification, delegation, and network-based support to improve access to neurological expertise. Originating from a single practice and later expanding into a multi-site medical care centre, the model now provides care for approximately 22,000 patients per quarter through an interdisciplinary team of medical assistants, physician assistants, and physicians [52, 53].

Its central principle is the systematic redistribution of tasks within a multiprofessional team. Standardised activities such as monitoring, coordination, documentation, patient education, and structured follow-up can be delegated to specifically trained non-physician staff [49, 53, 54]. In the context of MS care, this may support continuous general medical and psychosocial care, while allowing physicians and specialist partners to focus on diagnostic clarification, treatment decisions, escalation strategies, and complex or atypical disease courses. Importantly, the model remains embedded in a wider care network. In the present context, this includes immediate links to dedicated MS centres in Düsseldorf and Münster, enabling case discussion, specialist input, referral, or direct assessment whenever required. Community-based providers, outpatient specialists, and hospital-based partners can further contribute through telemedical consultation or direct assessment when needed. The broader relevance of the Papenburg model therefore lies not in replacing specialist care, but in creating a more resilient interface between local continuity of care and specialised expertise. It shows that rising care demands and workforce shortages may be addressed through role differentiation, structured delegation, and reliable links between primary, community-based, and specialist services.

The Papenburg model relies in particular on the integration of highly specialised and systematically trained physician assistants, whose delegated responsibilities are embedded within a structured multiprofessional care pathway. International experience suggests that physician assistants are an established component of team-based care in several healthcare systems. Particularly, in the United States and the Netherlands, physician assistants have been integrated into routine clinical care and workforce planning, and available evaluations indicate that, within clearly defined roles and supervised teams, they can support safe, acceptable and efficient care delivery. For German MS care, physician assistants may therefore represent one element of structured workforce redesign, helping to standardise recurring processes and preserve specialist physician capacity for complex diagnostic and therapeutic decisions. However, their broader integration remains an implementation challenge and will depend on the development of clear competency profiles, supervision standards, liability frameworks, and reimbursement mechanisms [49, 53, 54].

Several regional models also embed continuing education and clinical research directly into routine care delivery. Paderborn organises regular workshops and seminars for neurologists, general practitioners, and MS nurses. MS-Net in Mönchengladbach runs monthly immunoboards and roundtables with regional speakers, has established formal collaboration with patient organisations, and has implemented investigator-initiated clinical research, such as a study on sleep disorders, fatigue, and cognitive impairment in MS [55]. Together, these activities make the bridging element of “training and specialization of all care-involved professionals” identified in Fig. 1 operationally tangible at the regional level.

Beyond regionally embedded continuing education, structured qualification of MS specialists also requires formal, certifiable training programmes. A relevant example is the four-semester, part-time master’s programme “Multiple Sclerosis Management” (MSM), accredited in 2019 and offered at Dresden International University under the patronage of DMSG. It is the first academic degree programme designed around a single disease entity and is explicitly industry-independent, addressing physicians, therapists, nurses, scientists, pharmacists, psychologists, and biologists who wish to specialise in MS. The curriculum is organised into six modules including theoretical principles, clinical and diagnostic aspects, studies and statistics, DMTs, symptomatic therapy and rehabilitation, and monitoring and documentation, complemented by a master’s thesis, and combines lectures, tutorials, case conferences, journal clubs, and preceptorships in selected MS centres [56]. Delivered largely online through a cloud-based digital hub, the programme demonstrates how disease-specific expertise, practical competencies, and research literacy can be conveyed in a scalable, predominantly digital format. Such structured curricula are directly relevant to the delivery challenges described above: by building a shared knowledge base across professional groups and across the inpatient–outpatient interface, they support the skill-mix, delegation, and cross-sector coordination on which reproducible MS care depends.

#### Digital infrastructure for cross-sector MS care

Digital infrastructure is a prerequisite for scaling clinical and structural processes. Relevant elements include virtual case conferences, shared templates for safety monitoring and quality indicators, patient-reported outcome capture, symptom tracking, automated reminders, and escalation triggers [57]. Digital pathways should not be understood as stand-alone applications. Their value emerges when they are embedded in real clinical workflows, linked to responsibilities, and designed to reduce rather than increase documentation burden [42, 58–61].

Existing patient-generated and device-generated data could be used more effectively. Activity data from wearables, smartphones, or structured digital assessments may help identify changes between visits, support triage in rural regions, and complement questionnaire-based assessments that are vulnerable to recall bias. However, digital data require validation, governance, and clear thresholds for clinical action. Without these elements, digital monitoring risks producing data accumulation without better care [62].

To be effective, such digital infrastructure must be anchored in standardised, longitudinal monitoring concepts rather than in isolated tools. One example developed at the Center of Clinical Neuroscience in Dresden is a structured MS monitoring matrix, which organises the heterogeneous parameters of MS along two interrelated pathways - disease monitoring and intervention monitoring - and defines which parameters, instruments, and tools should be collected over time to enable adaptive, multimodal, and patient-centred care [17]. Building on this matrix, the concept of digital patient pathways translates these monitoring requirements into a modular-integrative framework that virtually links patients and caregivers, governs standardised data collection across sectors, and provides a foundation for advanced analytics, including digital twins [57]. To make the resulting care processes measurable, a consensus set of 24 quality indicators for the MS monitoring process was developed within the QPATH4MS project through a structured Delphi procedure with MS experts from Germany, Austria, and Switzerland. These indicators define a standardised monitoring work-up including encompassing general and neurological history, standardised examination, imaging, additional assessments, evaluation with recommended action, and work-up frequency and thereby operationalise the quality-indicator logic called for above [4]. Integrated into routine workflows, such pathways and indicators can render the coordination and monitoring work of complex MS care visible and auditable.

These elements converge in MS360°, a conceptual digital-first, data-driven hybrid care framework that integrates standardised on-site assessments with continuous remote monitoring, wearable sensors, and telemedicine. Rather than treating digital tools as stand-alone add-ons, MS360° uses continuously collected longitudinal data to dynamically steer structured patient pathways: predefined thresholds and patient profiles trigger targeted on-site assessments, teleconsultations, or escalation, while a multidisciplinary team reviews alerts, prioritises them by clinical urgency, and embeds documentation into existing systems. A tiered model – patient self-monitoring, automated flagging, and clinician intervention only for relevant alerts – is intended to optimise rather than increase workload, while quality indicators, dashboards, and iterative review cycles support continuous quality management. As such, MS360° illustrates how the monitoring matrix, digital patient pathways, and consensus quality indicators can be combined into an implementation-ready hybrid care model that directly addresses the fragmentation, episodic monitoring, and access constraints described above, and provides a transferable building block for the cross-sector and hub-and-spoke architectures discussed in this review [60].

#### Innovation Fund projects as test beds for digital and cross-sector care

The Innovation Fund (Innovationsfonds) of the G-BA is a health-policy instrument designed to test innovative approaches in statutory health insurance and to generate routine-care evidence that can inform future regulation. It supports two streams of direct relevance for MS care: Versorgungsforschung, which investigates care patterns, access, and outcomes, and Neue Versorgungsformen, which pilots service models beyond the standard catalogue. Successful models can subsequently be transferred into regular reimbursement.

Several projects have addressed MS-relevant questions and share a common goal of improving access, coordination, and patient experience: digital and telemedical follow-up after diagnosis, structured shared decision-making at treatment initiation, integration of patient-reported outcomes into routine care, and registry-based long-term safety surveillance. Examples include MSnetWork (funding ID 01NVF20025), VerSI-MS-PV (funding ID 01VSF18038), and KOKOS-MS (funding ID 01VSF19029). Taken together, these projects test exactly the building blocks discussed earlier in this review: digitalisation, intersectoral collaboration, and skill-mix.

A current, particularly relevant example for low-density regions and area-wide care is TENEAM (TeleNeurologisch ambulante Versorgung in Brandenburg und Mecklenburg-Vorpommern; funding ID 01NVF24001) [63]. TENEAM goes beyond established telemedical follow-up: it also processes first patient contacts through a structured telemedical pathway. This matters because long waiting times for neurology appointments are one of the main barriers to early diagnosis in rural regions. A telemedical first contact can triage patients more rapidly, accelerate scheduling of MRI and specialist evaluation, and shorten the interval between symptom onset and confirmed diagnosis, particularly where the nearest MS-experienced neurologist is hours away by car.

Such projects matter for two reasons. First, they create an evaluation framework for care models that are conceptually plausible but require controlled evidence on feasibility, safety, patient acceptance, and transferability before they can be considered for inclusion in regular reimbursement. Second, they shift the unit of innovation from the individual practice to the regional system: telemedical first-contact pathways, virtual MS boards, or shared documentation pipelines are only meaningful when several actors agree on common rules.

#### Living Labs as engines of digital MS care innovation

While the Innovation Fund provides a regional test bed for whole care models, a complementary mechanism is needed for the earlier, faster cycles in which individual digital tools are co-designed, tested, and iteratively refined. Living Labs offer such a mechanism: clinic-embedded, co-designed environments in which MS patients, clinicians, developers, and regulators jointly develop and evaluate digital health technologies in near-real-world conditions. Rather than replacing clinical trials, Living Labs adapt the rigorously managed trial environment into a more flexible setting, allowing safe exploration of varied uses, workflows, and device–human interactions across technology readiness levels. This addresses a recurrent bottleneck in MS care, where promising digital biomarkers, apps, and AI-based tools often remain confined to isolated pilot projects because of regulatory hurdles, fragmented infrastructure, and a lack of agile real-world evaluation methods. Crucially, the flexibility of a Living Lab does not imply an “anything goes” approach: it depends on continuous digital and ambient monitoring, clearly defined standard operating procedures, dynamic consent, and a dedicated quality and ethics function, so that adaptive iteration remains auditable and compliant with frameworks such as the MDR, GDPR, and the EU AI Act.

When embedded within a dedicated MS centre, a Living Lab couples naturally with the learning health system logic described above: short cycles of data capture, quality checks, and outcome review feed back into standard operating procedures and tool design, with results written to the electronic health record under auditable standards. The MS Living Lab established at the ZKN Dresden illustrates this concept, embedding high-resolution assessments directly into the neurological examination and extending data collection into patients’ daily lives, with a clinic-integrated speech module (MSPEECH) as one co-designed exemplar. By providing a protected, quality-controlled space for iterative development and feasibility testing and by linking to complementary instruments such as in-house device development and regulatory sandboxes – Living Labs can shorten the path from a promising prototype to a validated, scalable tool. In this way, they act as an innovation engine for hybrid MS care, generating the standardised, real-world evidence on feasibility, data quality, usability, and clinical utility that the digital pathways, quality indicators, and future digital twins described above ultimately depend on.

#### Digital twins and artificial intelligence as future components

Digital twins and artificial intelligence may become future components of digital MS care pathways but should be framed as downstream decision-support tools rather than as near-term replacements for clinical judgement. In principle, an AI-based synthesis of clinical, imaging, laboratory, functional, and patient-generated data can create a patient-specific digital representation that supports risk prediction, patient communication, shared decision-making, and therapy refinement. The central requirements are validation, interpretability, data quality, privacy, and integration into clinical accountability [64, 65].

A digital twin for MS (DTMS) has been proposed as a conceptual endpoint of this development: a virtual, continuously updated representation of the individual patient generated through AI-based analysis of clinical and para-clinical outcomes, multi-omics and biomarker data, imaging, patient-reported data, and information on the patient’s life circumstances. Such a twin would integrate data from multiple sources in a standardised manner, embed them into personalised clinical pathways, and present pre-analysed information on a dashboard to support physician–patient communication, shared decision-making, prediction of disease progression, and treatment simulation. Conceived as a learning health system, the DTMS would continuously incorporate newly collected real-world data and refine its predictions over time. Importantly, the DTMS is more than a data-collection or rule-based clinical decision-support approach: by correlating dynamic, multidimensional data and feeding them into simulation processes with defined clinical and economic goals, it could allow therapies to be tested in silico before application and thereby support precision medicine and patient-centred care [66]. Realising this vision, however, depends on the same prerequisites that govern hybrid care more generally – high-quality, standardised, multicentre data over many years, robust governance for data privacy and security, attention to algorithmic bias and explainability, and prospective clinical validation – underscoring that the digital infrastructure, standardised monitoring, and quality indicators described above are not merely operational details but the foundation on which any future MS digital twin must be built.

### Hub-and-spoke networks as a scalable care architecture

Taken together, the preceding examples suggest that scalable MS care requires more than individual regional initiatives. ASV-MS, complex care pathways, delegated team structures, teaching networks and digital infrastructures address specific parts of the same delivery problem: how to make specialised MS expertise available beyond highly resourced centres without diluting clinical quality. Hub-and-spoke care provides a conceptual architecture for integrating these elements into a reproducible regional model.

In such a model, the hub does not replace local neurological care, but concentrates tasks that require high-level expertise, including diagnostic confirmation, initiation of high-complexity therapies, risk stratification, treatment switches, complex de-escalation decisions and multidisciplinary case conferences. Spokes provide routine follow-up, defined safety monitoring, patient education, relapse assessment and supportive care under shared protocols. Digital infrastructure connects both levels through shared documentation, virtual MS boards, escalation triggers and agreed quality indicators, while trained non-physician roles support monitoring, coordination and patient guidance.

**Fig. 2:**
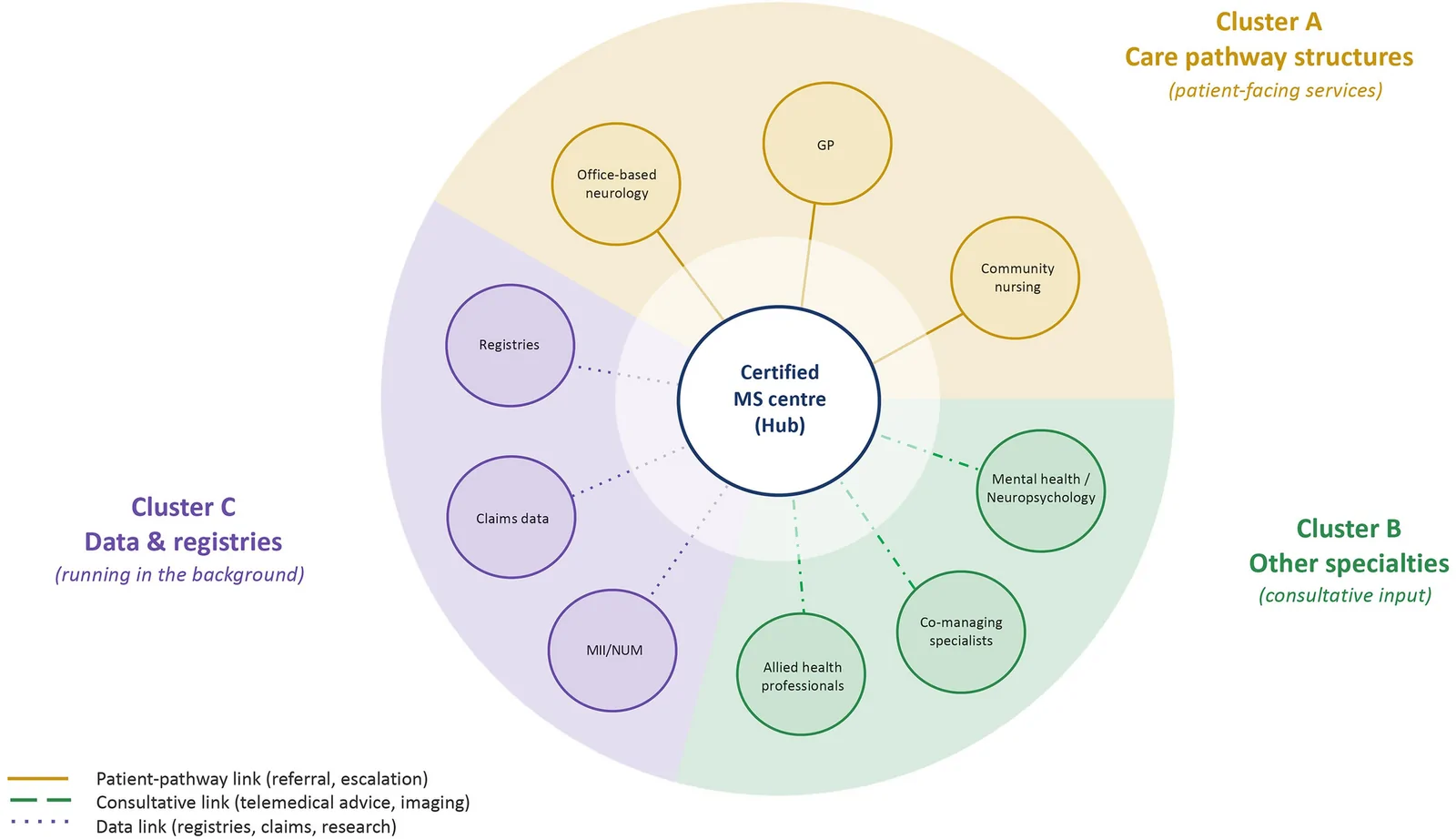
Hub-and-spoke MS care pathway with shared monitoring roles

This architecture is particularly relevant for rural or low-density regions, where full specialist capacity cannot be replicated at every site. Rather than adding another isolated example, hub-and-spoke care integrates the implementation pathways discussed above into a scalable regional model. The architecture itself is not specific to the German healthcare system, although the mechanisms through which it can be implemented and reimbursed will vary substantially between healthcare settings. Specialised centres concentrate complex decision-making, regional partners provide longitudinal and supportive care, and training as well as digital infrastructure connect both levels. Figure 2 summarises this shared-care logic.

The figure illustrates a regional hub-and-spoke model for integrated MS care, centred around the MS patient and coordinated through a certified MS centre acting as the hub. Patient-facing care pathway structures include office-based neurology, general practitioners, community nursing, allied health professionals, mental health and neuropsychology services, and co-managing specialists. These actors are connected to the hub through patient-pathway links, including referral and escalation pathways. Additional specialist input can be integrated through consultative links, including telemedical advice and imaging-based support. Data and registry structures, including registries, claims data, and research infrastructures such as MII and NUM, run in the background and provide a complementary layer for quality assurance, benchmarking, health-services research, and care planning. Together, the model highlights that scalable MS care depends not only on specialist centres, but also on reliable interfaces between local care, specialist consultation, and shared data infrastructure. Abbreviations: GP, general practitioner; MII, Medical Informatics Initiative; MS, multiple sclerosis; NUM, Netzwerk Universitätsmedizin.

### Implementation priorities and future directions

The regional examples summarised above show that the limiting step in German MS care is no longer the absence of plausible solutions, but the absence of mechanisms to translate them into reproducible care structures across heterogeneous settings. Rather than promoting a single preferred model, the more relevant task is to specify the structural, organisational and financial conditions under which different regional approaches can deliver equitable, safe, and sustainable MS care. This requires an implementation agenda that links measurable quality, workforce redesign, digital infrastructure, and health-policy support, the four levers most likely to convert isolated regional innovation into durable national capacity.

A first priority is the implementation of consensus-based quality indicators that make service delivery visible beyond therapeutic intent alone. At the regional level, such indicators could capture time to diagnosis, time to treatment decision, time to DMT initiation, MRI monitoring intervals, adherence to safety laboratory schedules, relapse-management pathways, the proportion of patients without DMTs, and access to rehabilitation, cognitive screening, or specialised review. Their purpose is not administrative control, but system learning: identifying bottlenecks, comparing delivery models, and informing targeted resource allocation [4, 15, 67–69].

A second priority is to make the coordination work that complex MS care depends on financially and structurally visible. Virtual case discussions, shared documentation, therapy-switch planning, radiology interfaces, patient education, and cross-sector communication are clinically essential, yet they typically appear neither in service descriptions nor in reimbursement codes. Similar coordination efforts are embedded within multidisciplinary neurological complex treatment programmes, where complex medical decision-making, interdisciplinary treatment planning, and coordinated transition back into outpatient care are integrated into a defined episode of specialised care. When developing future regional MS care models and reimbursement frameworks, these programmes should therefore be recognised as complementary structural components of specialised MS care alongside ambulatory specialist care, rehabilitation, and digital care models, rather than being viewed solely as inpatient therapeutic interventions. Unless coordination-intensive care structures are adequately reflected in service models and reimbursement logic, many regional networks will continue to be dependent on individual commitment rather than durable infrastructure [70].

Third, delegation and role redesign need to be formalised. Scalable models require explicitly defined competencies, structured supervision, and accredited training pathways for MS nurses, advanced medical assistants, and physician assistants [35, 49]. This is especially relevant for high-efficacy treatment pathways, where protocol adherence, patient counselling, and timely recognition of safety signals are critical.

Finally, digital tools should be judged by whether they improve care delivery rather than by their technical novelty. Interoperable templates, patient-reported outcomes, wearable-derived activity data, and decision-support tools are most valuable when linked to concrete clinical questions, thresholds, and responsibilities [57]. Future evaluation should therefore focus on outcomes such as access, waiting times, treatment initiation, monitoring completeness, hospitalisations, patient and caregiver burden, staff workload, safety events, and cost consequences. This will help to distinguish transferable core components from those that are primarily context-specific [71].

### Limitations

This review has several limitations. First, the literature search was non-systematic and therefore may not have captured all relevant evidence or regional care models. Second, no formal evidence grading or systematic assessment of study quality was performed, as the aim was to provide a delivery-oriented synthesis rather than a systematic evidence review. Third, the selection of regional models was purposive and partly informed by expert knowledge, including the authors’ involvement in several of the initiatives described. Finally, the heterogeneity of regional healthcare structures and reimbursement frameworks limits the generalisability and direct transferability of individual models to other settings.

## Conclusion

Modern MS care in Germany is increasingly shaped not only by therapeutic advances, but by the system’s ability to deliver them consistently, safely, and close to the patient. Regional variation in treatment uptake, monitoring, and access indicates that care capacity, coordination, and workforce structure directly influence clinical practice. The central task for the coming years is therefore not simply to expand specialist care, but to organise it more intelligently - through shared-care arrangements, delegated team structures, measurable quality standards, and digital pathways that bring specialist expertise into routine care without overextending it. For neurology, this means moving from isolated regional innovation to reproducible implementation models that preserve specialist judgement while making high-quality MS care more equitable across regions.

## Data Availability

No datasets were generated or analysed during the current study.

## Abbreviations

ASV: Ambulante spezialfachärztliche Versorgung (specialized outpatient care)
AWMF: Arbeitsgemeinschaft der Wissenschaftlichen Medizinischen Fachgesellschaften (Association of the Scientific Medical Societies)
DMT: Disease-modifying therapy
DMSG: Deutsche Multiple Sklerose Gesellschaft (German Multiple Sclerosis Society)
DRG: Diagnosis-related group
DTMS: Digitales Therapie- und Monitoring-System (Digital Therapy and Monitoring System)
EBM: Einheitlicher Bewertungsmaßstab (Uniform Value Scale)
EMA: European Medicines Agency
G-BA: Gemeinsamer Bundesausschuss (Federal Joint Committee)
GDPR: General Data Protection Regulation
GdB: Grad der Behinderung (degree of disability)
GP: General practitioner
HAS: Hochschulambulanz (university outpatient clinic)
IV: Integrated care
KKNMS: Kompetenznetz Multiple Sklerose (German Competence Network Multiple Sclerosis)
KV: Kassenärztliche Vereinigung (Association of Statutory Health Insurance Physicians)
LMU: Ludwig-Maximilians-Universität München (Ludwig Maximilian University of Munich)
MDR: Medical Device Regulation
MII: Medizin-Informatik-Initiative (Medical Informatics Initiative)
MS: Multiple sclerosis
MZEB: Medizinisches Zentrum für Erwachsene mit Behinderung (Medical Centre for Adults with Disabilities)
NeNET: Neurology Network
NUM: Netzwerk Universitätsmedizin (Network of University Medicine)
QPATH4MS: Quality Pathways for Multiple Sclerosis
SGB V: Sozialgesetzbuch V (German Social Code Book V)
WHO: World Health Organization
